# Insights on Covid‐19 with superimposed pulmonary histoplasmosis: The possible nexus

**DOI:** 10.1002/iid3.989

**Published:** 2023-09-29

**Authors:** Yahya A. Almutawif, Hayder M. Al‐kuraishy, Ali I. Al‐Gareeb, Athanasios Alexiou, Marios Papadakis, Hamza M. A. Eid, Hebatallah M. Saad, Gaber El‐Saber Batiha

**Affiliations:** ^1^ Department of Medical Laboratories Technology, College of Applied Medical Sciences Taibah University Madinah Saudi Arabia; ^2^ Department of Clinical Pharmacology and Medicine, College of Medicine Al‐Mustansiriyia University Baghdad Iraq; ^3^ Department of Science and Engineering Novel Global Community Educational Foundation Hebersham New South Wales Australia; ^4^ AFNP Med Wien Austria; ^5^ Department of Surgery II, University Hospital Witten‐Herdecke, Heusnerstrasse 40 University of Witten‐Herdecke Wuppertal Germany; ^6^ Department of Pathology, Faculty of Veterinary Medicine Matrouh University Marsa Matruh Egypt; ^7^ Department of Pharmacology and Therapeutics, Faculty of Veterinary Medicine Damanhour University Damanhour AlBeheira Egypt

**Keywords:** Covid‐19, inflammatory signaling pathways, pulmonary histoplasmosis

## Abstract

A novel coronavirus (CoV) known as severe acute respiratory syndrome CoV type 2 is the causative agent for the development of CoV disease 2019 (Covid‐19). Covid‐19 may increase the risk of developing pulmonary histoplasmosis due to immune dysregulation. In addition, Covid‐19 may enhance the propagation of acute pulmonary histoplasmosis due to lung injury and inflammation, and using corticosteroids in severely affected Covid‐19 patients may reactivate latent pulmonary histoplasmosis. Likewise, activation of inflammatory signaling pathways during *H. capsulatum* infection may increase the severity of Covid‐19 and vice versa. Furthermore, lymphopenia in Covid‐19 may increase the risk for the progress of pulmonary histoplasmosis besides activation of inflammatory signaling pathways during *H. capsulatum* infection may increase the severity of Covid‐19 and vice versa. Therefore, this critical review aimed to find the potential link between Covid‐19 pneumonia and pulmonary histoplasmosis concerning the immunological response.

## Graphical abstract

Pulmonary histoplasmosis may occur in Covid‐19 patients; therefore, immune dysregulation chiefly lymphopenia in Covid‐19 may increase the risk for the progress of pulmonary histoplasmosis. As well, the activation of inflammatory signaling pathways during H. Capsulatum infection may increase the severity of Covid‐19 and vice versa.

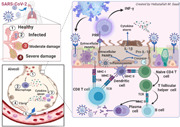

## 1 INTRODUCTION

A novel coronavirus (CoV) known as severe acute respiratory syndrome CoV type 2 (SARS‐CoV‐2) was primarily identified as the potential cause of acute respiratory infection in Wuhan, Hubei province of China, in late December 2019.^1^ In early 2020, the World Health Organization designated this disease as coronavirus disease 2019 (Covid‐19).^2^ Angiotensin‐converting enzyme type 2 (ACE2) is one of the most prevalent receptors SARS‐CoV‐2 uses to enter human cells.^3^ When SARS‐CoV‐2 binds to ACE2, several inflammatory cellular processes with cytopathic effects occur, resulting in cellular damage and inflammation. Numerous cellular systems, such as enterocytes, cardiomyocytes, lung alveolar cells, neurons, and testes, are the primary sites of distribution and expression of ACE2.^4^

In 85% of patients, Covid‐19's clinical manifestation is primarily asymptomatic or has minor symptoms.^5^ Due to the development of acute lung injury (ALI), 15% of patients presented with a moderate‐to‐severe type. Additionally, 5% of Covid‐19 patients may need assisted breathing because of the emergence of acute respiratory distress syndrome (ARDS).^6^

SARS‐CoV‐2 shares 80% and 60% of the genetic similarities with other CoVs like SARS and Middle East Respiratory Syndrome CoV (MERS‐CoV), respectively. Additionally, SARS‐CoV‐2 shares 96% of its genomic sequence with the bat CoV. Although SARS‐CoV‐2 uses and binds ACE2 20 times more frequently than other CoVs, these receptors are subsequently downregulated.^7^ As a result, Angiotensin II (Ang II), a vasoconstrictor, is converted by the enzyme ACE2 into the vasodilators Ang1‐7 and Ang1‐9. As a result, the SARS‐CoV‐2 infection causes vasoconstriction and develops inflammatory diseases, oxidative stress, and endothelial dysfunction.^8^

Histoplasmosis is a fungal infection caused by *Histoplasma capsulatum* (*H. capsulatum*), a dimorphic intracellular fungus that causes systemic and respiratory infections in both immunocompetent and immunocompromised subjects.^9^ Histoplasmosis was initially discovered by Darling among the workers of the Panama Canal in 1960. Histoplasmosis is regarded as the most common cause of respiratory fungal infection, with 500,000 cases annually.^10^ Histoplasmosis is the most common prevalent disease among individuals in Ohio and Mississippi, United States, and it has been shown that 80% of living subjects are seropositive for *H. capsulatum*.^10^ Immune disorders in Covid‐19 may increase the risk of developing pulmonary histoplasmosis.^11^, ^12^, ^13^ Therefore, this review aimed to find how Covid‐19 increases pulmonary histoplasmosis concerning the immunological response.

## 2 PULMONARY HISTOPLASMOSIS AND IMMUNE RESPONSE

*H. capsulatum* is mainly found in the soil in a mycelia form and enters into hosts like humans via inhalation of microconidia.^14^ Neutrophils and macrophages engulf *H. capsulatum* in the respiratory epithelium; some inhaled *H. capsulatum* is rapidly transported by lymphatics to the bloodstream to avoid their destruction by the immune cells.^14^ Body temperature increases the differentiation of *H. capsulatum* into pathogenic yeast, which survives within the phagocytes.^15^ Many intracellular signaling pathways, like histidine kinase, regulate yeast proliferation expression and immune cell recognition.^16^ Typically, pulmonary histoplasmosis is controlled by innate and adaptive immune responses; therefore, this disease may be asymptomatic or presented with flu‐like illness in immunocompetent subjects.^17^ Of note, acute pulmonary histoplasmosis occurs in outbreaks leading to symptomatic diseases in specific individuals. However, in immunocompromised patients or individuals with low CD4 T cells subjected to high inoculums, severe disease may develop with a high fatality risk, even in patients treated with adequate antifungal agents.^18^

### 2.1 Virulence factors

*H. capsulatum* has different virulence factors, such as surface molecules mediating the interaction between this pathogen and immune cells and promoting pathogen evasion from destruction during the innate immune response.^19^ *H. capsulatum* can utilize host macrophages as a niche for proliferation. Thus, the innate immune response is not adequate to prevent the development of infection with *H. capsulatum*.^19^ However, activated dendritic cells promote immune response through the expression of toll‐like receptors (TLRs) and polarization toward type 1 immune response (Th1) with subsequent release of type 1 interferon (IFN‐1).^20^ Th1 immune response increases clearance of *H. capsulatum* through the recruitment of neutrophils by the effect of IL‐17. Though, the Th2 immune response drives the allergic response to *H. capsulatum* via the production of IL‐5 and the recruitment of eosinophils.^20^ Moreover, heat shock protein 60 (HSP60), which regulates intracellular protein folding, is regarded as an important surface molecule that mediates phagocytosis of *H. capsulatum* by the activated macrophages.^21^ HSP60 of *H. capsulatum* protects against antibody response and improves biofilm formation.^21^ HSP60 acts as a ligand for macrophage receptors during fungal infection, though this interaction induces a mild host immune response.^22^ In this state, *H. capsulatum* replicates and survives within the host cells. As well, HSP82 participates in the immune response through the inactivation of different cellular proteins and regulation of activation signaling. *H. capsulatum*, through induction of temperature and cellular stress, promotes HSP82 expression, reducing the virulence of *H. capsulatum* within the macrophages.^23^ As well, HSP82 maintains and preserves body temperature and cellular function, respectively, during the development of oxidative stress.^23^ In addition, *H. capsulatum* expresses α‐glucan on the cell wall that hides β‐glucan, which has an antigenic effect identified by immune cells. The deficiency of α‐glucan in *H. capsulatum* is linked with low virulence and rapid immune response.^24^ Also, histones and fungal hydroxamate siderophores act as virulent factors and are implicated in immune response activation.^25^ Moreover, the virulence of *H. capsulatum* is associated with the production of melanin and melanin‐like pigments. The melanization process reduces the susceptibility of *H. capsulatum* to the effects of antifungal agents through attenuation of the host defense mechanism.^26^, ^27^ Liu et al.^28^ observed that fungal melanin impairs phagocytic functions by reducing antibody and immune responses through complex interactions with molecules of immune cells. Furthermore, the pathogenesis of *H. capsulatum* is correlated with the secretion of calcium‐binding protein (CBP) released from *H. capsulatum* during the yeast phase inside the activated macrophages both in vivo and in vitro.^29^ Deleting the CBP gene promotes clearance of fungal infection in the experimental mice.^29^ Moreover, only three antigens specifically recognized by antibodies of histoplasmosis patients were mapped as potential antigenic targets: the M antigen, previously demonstrated in the diagnosis of histoplasmosis, and the catalase P and YPS‐3 proteins, characterized as virulence factors of *H. capsulatum*, with antigenic properties still unclear. The other two proteins were fragments of the YPS‐3 and M antigen. Overlapping results obtained from the two aforementioned bioinformatic tools, 16 regions from these three proteins are proposed as putative B‐cell epitopes exclusive to *H. capsulatum*. These data reveal a new role for these proteins on *H. capsulatum* interactions with the immune system and indicate their possible use in new methods for the diagnosis of histoplasmosis.^30^ Moreover, antibodies against the M and H antigens, a catalase B and beta‐glucosidase, respectively, are consistently produced by patients with different clinical forms of histoplasmosis and, therefore, are largely applied in serodiagnostic tests.^31^ YPS‐3 protein, localized in the cell wall and culture supernatants of *H. capsulatum*, may influence the dimorphic transition. The *yps‐3* gene expression is not fundamental for the transformation to the yeast phase, but it may facilitate adaptive processes that allow mycelium‐to‐yeast transition and survival at elevated host temperatures. Moreover, YPS‐3 has already been associated with increased fungal burden in phagocyte‐rich tissues.^32^ The YPS‐3 protein, present in the cell wall and also released in culture medium, is produced only by the yeast phase of *H. capsulatum*.^33^ Its location suggests that YPS3 is a protein capable of being recognized by antibodies and the results of the present study show for the first time the antigenic potential of this protein. A previous study proposed YPS3 to be virulence factor involved in the progression of the disseminated disease, since, when its production was blocked by interference RNA in animal infection, the mutants showed a significant decrease in fungal dissemination.^32^

These observations indicated that HSP60, HSP82, α‐glucan, hydroxamate siderophores and fungal melanin are regarded as the most important virulence factor of *H. capsulatum* in the pathogenesis of pulmonary histoplasmosis.

### 2.2 Immune response

During infection with *H. capsulatum*, the innate and adaptive immune systems are both activated to neutralize the invading pathogen. Dendritic and macrophages have critical roles in stimulating cellular pathways and releasing proinflammatory cytokines in infection with *H. capsulatum*. Activated macrophages trigger the activation of the TH1 immune response and release of proinflammatory cytokines and IFN‐γ.^34^ However, *H. capsulatum* and other fungi can evade phagocytosis‐induced destruction by neutralizing intracellular pH and phagolysosomes and inhibiting lysosomal hydrolase.^35^ Nevertheless, dendritic and macrophages can recognize, degrade and phagocytize fungal cells through expressed receptors on the dendritic and macrophages.^35^ During activation of the innate immune response, dendritic cells play a potential role in presenting antigens of *H. capsulatum* to the CD8 T cells, which have a central role in regulating cellular immunity during pulmonary histoplasmosis.^36^ It has been illustrated that lymphopenia was associated with more complications and mortality in the experimental mice with pulmonary histoplasmosis.^37^ Lymphopenia may differentially affect the course of fungal infections. For example, depletion of CD4 T cells during primary fungal infections promotes survival, whereas depletion of CD8 T cells attenuates fungal clearance in the primary infection. Remarkably, Vβ4+ T cells which are CD8 T cell subtypes are preferentially activated during pulmonary histoplasmosis, and contribute to the generation of protective immunity by recognizing HSP60 from *H. capsulatum*, and the elimination of these immune cells prevents the resolution of this infection in mice.^38^, ^39^ Progressive disseminated pulmonary histoplasmosis is most commonly recognized in AIDS patients, idiopathic lymphocytopenia, and common variable immunodeficiency.^39^ An observational study illustrated that pulmonary histoplasmosis was common in patients with lymphoma and leukemia treated with bendamustine chemotherapy.^38^ Over‐activation of Th17 and reduction of regulatory T cells (Treg) are associated with the acceleration and clearance of fungal infections, including *H. capsulatum*.^40^ Chemotactic mediator CCR5 of Treg and proinflammatory T cells are involved in eradicating *H. capsulatum* infection.^41^ In an experimental study, lacking CCR5 attenuated infiltration of T cells to the pulmonary tissues in mice with pulmonary histoplasmosis.^40^ As well, the reduction of circulating Treg is associated with acceleration of *H. capsulatum* infection.^40^

On the other hand, cytokine response in *H. capsulatum* infection is complex; IL‐12 promotes the primary immune response by inducing the release of IFN‐γ, which increases clearance of *H. capsulatum* infection.^42^ Impairment of IFN‐γ in *H. capsulatum* infection increases the risk of developing severe complications.^42^ Zerbe et al.^43^ found that patients with mutations in IFN‐γ receptors had a higher risk of developing severe *H. capsulatum* infection. In addition, antibody production and generation of reactive oxygen species have little role against *H. capsulatum* infection, as disseminated histoplasmosis was not developed in patients with chronic granulomatous disease and agammaglobulinemia.^43^ In contrast, the severity of disseminated histoplasmosis is correlated with deficiency of CD40 ligand in patients with lymphopenia.^43^ Therefore, lymphopenia and immunosuppression increase the risk of developing severe infection with *H. capsulatum*.

Of interest, proinflammatory cytokines like TNF‐α promote clearance of *H. capsulatum* and reduce infection severity. It has been reported that administering anti‐TNF‐α inhibitors augments the development of severe disseminated histoplasmosis with an increased risk for reactivation of latent histoplasmosis.^22^, ^44^ Wood and co‐workers illustrated that using anti‐TNF‐α inhibitors infliximab enhances *H. capsulatum* infection in patients with chronic inflammatory disorders due to the inhibition of monocytes and alveolar macrophages with attenuation of cellular immunity.^45^

Conversely, the humoral immune response is limited in the control and clearance of *H. capsulatum* infection. However, administering specific antibodies against surface molecules of *H. capsulatum* may reduce the propagation of pulmonary histoplasmosis in murine models.^46^ Similarly, administering monoclonal antibodies against *H. capsulatum* HSP60 reduces the risk of disease severity.^47^ Recently, Moura et al.^48^ found that chimeric mouse‐human monoclonal antibody against *H. capsulatum* HSP60 produces a protective effect in mice with experimental disseminated histoplasmosis. Furthermore, vaccines and monoclonal antibodies also modulate the *H. capsulatum* membrane composition of phosphatidylinositol, phosphatidylethanolamine, and phosphatidylcholine with subsequent reduction response to the antifungal agents.^49^

**Figure 1:**
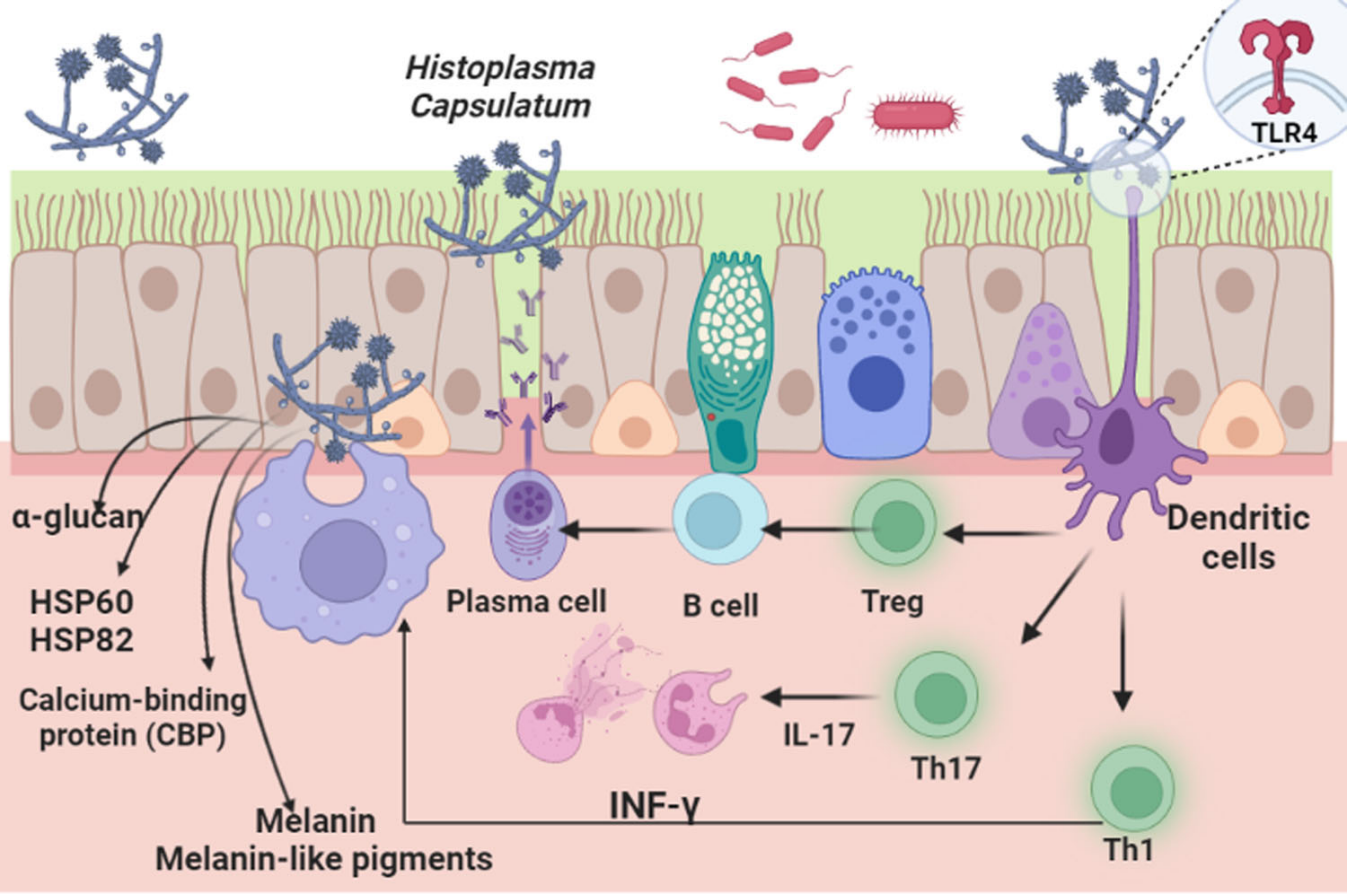
Pulmonary histoplasmosis and immune response.

Taken together, activation of the immune response during *H. capsulatum* infection mainly depends on the modulation of IFN‐γ release and activation of cellular immunity. However, humoral immune response and production of antibodies have a minor role in controlling pulmonary histoplasmosis (Figure 1).

## 3 COVID‐19 AND IMMUNE RESPONSE

The human immune response to the SARS‐CoV‐2 infection is typically started when the infected cells express pathogen‐associated molecular patterns (PAMPs), which are recognized by pattern recognition receptors (PRR).^50^ Toll‐like receptor 4 (TLR4) is one of the most important PRRs expressed by neutrophils, macrophages, and monocytes, identifying extracellular PAMPs.^6^ Stimulated TLR4 triggers the release of IFN‐γ, which has a potent antiviral effect by recognizing extracellular PAMPs.^6^ The intracellular danger‐associated molecular patterns are identified by cytosolic sensors like node‐like receptor pyrin 3 (NLRP3) inflammasome. In this state, NLRP3 inflammasome triggers activation of pro‐caspase 1, which increases the conversion of pro‐IL‐1β to IL‐1β.^51^ IL‐1β involved in the propagation of hyperinflammation and cytokine storm.^52^

In general, SARS‐CoV‐2‐infected cells are recognized and destroyed by natural killer cells of innate immunity and CD8 of adaptive immunity through the perforin‐linked process with induction of apoptosis.^7^ In this immunological interaction and to avoid abnormal immune hyperactivation, CD8 and antigen‐presenting cells undergo apoptosis following this order.^8^ Remarkably, if there is a defect in helper T cells, the natural killer and CD8 T cells cannot destroy and remove virally infected cells resulting in intense immunological interaction and exaggerated innate and adaptive immune responses.^53^, ^54^

**Figure 2:**
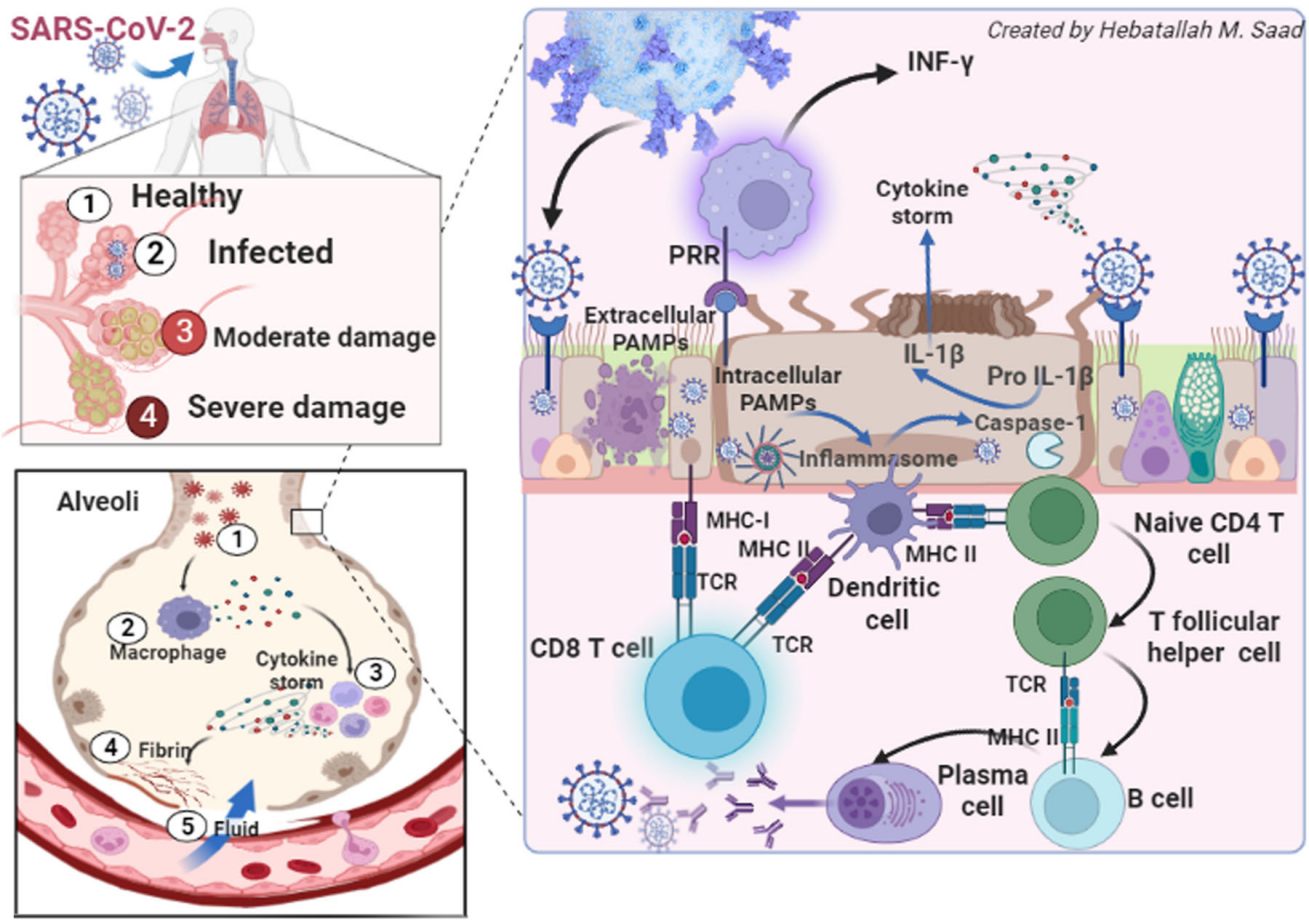
Covid‐19 and immune response.

A prolonged immune response induces an exaggerated immune response and release of proinflammatory cytokines causing hyperinflammation, hypercytokinemia, and the development of cytokine storm.^55^ As well, imperfection of lymphocyte functions induces uncontrolled macrophage activation and propagation development of macrophage activation syndrome (MAS), characterized by the release of a considerable amount of proinflammatory cytokines and the progress of hyperinflammation.^56^ Defects in the clearance of SARS‐CoV‐2, attenuation of IFN‐γ response, pyroptosis, ferroptosis, and formation of neutrophil extracellular traps could be the potential mechanisms behind the development of MAS in Covid‐19.^57^ Therefore, as SARS‐CoV‐2 can evade the immune response, it may lead to a defect in the interaction between innate and adaptive with subsequent exaggerated immune response. Notably, direct SARS‐CoV‐2 cytopathic and exaggerated immune response interacts mutually in ALI/ARDS and multiorgan failure (MOF) progression in patients with severe Covid‐19.^58^ Interestingly, SARS‐CoV‐2 triggers an antiviral immune response and may lead to the development of an exaggerated immuno‐inflammatory response with the propagation of lymphocyte dysfunction, lymphopenia, and neutrophil abnormalities.^59^, ^60^ Herein, inhibition of systemic inflammation could be a promising therapeutic strategy to prevent tissue injury in severe Covid‐19^60^ (Figure 2).

### 3.1 Covid‐19 and lymphopenia

Lymphopenia is a crucial laboratory feature of Covid‐19, mainly in severe cases. Developing lymphopenia is linked to increased intensive care unit (ICU) admissions and adverse medical outcomes.^6^ In patients with severe Covid‐19, the lymphocyte percentage is reduced to less than 20% compared to the mild form.^6^ CD8 T cells and memory helper T cells are also mainly reduced in patients with severe Covid‐19.^61^ These observations proposed that lymphopenia is regarded as a potential indicator of Covid‐19 severity.

In contrast, the number of B cells is still within the normal range, even in severe Covid‐19 patients. Therefore, lymphopenia in severe Covid‐19 patients may result from over‐activation and exhaustion of T cells. Notably, T‐cell activation was reported to be linked with Covid‐19 pathogenesis. For example, Li et al.^62^ found that T cell activation, mainly CD8, was associated with SARS‐CoV infection. Likewise, the expression of CD38, CD69, and CD44 on CD4 and CD8 T cells is increased in severe Covid‐19 patients compared to the mild one.^63^ In addition, the expression of 4‐1BB and OX40, key molecules for priming immune response and clonal expansion, are highly augmented in severe Covid‐19 patients.^63^ Amusingly, biomarkers of T cell exhaustion like mucin domain 3, T cell immunoglobulin domain, and programmed cell death protein 1 are augmented in severe Covid‐19 patients.^64^ Therefore, the reduction of T cell functional diversity and increasing biomarker levels of T cell exhaustion indicate the progression of Covid‐19 to the severe form.^64^

The underlying mechanisms of lymphopenia in severe Covid‐19 are related to direct cytopathic injury of lymphocytes through the ACE2‐mediated pathway, hypercytokinemia with the development of cytokine storm, which promotes exhaustion and depletion of T cells. Similarly, SARS‐CoV‐2 may injure the lymphoid organs causing lymphopenia in Covid‐19. Of note, suppression of lymphocyte proliferation in Covid‐19 might develop due to increasing levels of lactic acid.^65^, ^66^, ^67^ Therefore, restoration of the anti‐inflammatory function of lymphocytes by inhibiting SARS‐CoV‐2‐induced lymphopenia and associated hyperinflammation could be considered an effective therapeutic modality against developing Covid‐19 severity.

## 4 COVID‐19 AND PULMONARY HISTOPLASMOSIS

Covid‐19 patients are at high risk for fungal infections such as candidiasis and invasive pulmonary aspergillosis, increasing morbidity and mortality.^68^ Different studies highlighted the association between Covid‐19 and pulmonary histoplasmosis (Table 1). *H. capsulatum* infection or latent reactivation must be considered in Covid‐19 patients living in endemic areas. A cohort study included 39 Covid‐19 patients without a history of AIDS and revealed that sample plasma detected anti‐*H. capsulatum* antibodies in 20.51% of patients.^68^ However, a retrospective study involving 836 Covid‐19 patients showed no clinical evidence of fungal infection in the early and late phases of Covid‐19 in critically hospitalized patients.^69^ Blood cultures, respiratory samples, pneumococcal or *Legionella* urinary antigens and respiratory viral PCR panels were obtained from those patients, found a low frequency of bacterial coinfection in early Covid‐19 hospital presentation, and no evidence of concomitant fungal infection, at least in the early phase of Covid‐19.^69^ This study depended on the fungal culture which not excludes fungal infections. A case study reported that a 61‐year‐old man was admitted to the hospital due to Covid‐19 pneumonia, and despite appropriate management, the patient was transferred to ICU for mechanical ventilation due to the development of respiratory failure. After 2 weeks, co‐infection with pulmonary histoplasmosis was detected, which was treated by itraconazole and amphotericin B, and the patient dramatically improved.^70^ Furthermore, anti‐TNF‐α antagonist tocilizumab, effectively used in Covid‐19 patients to prevent cytokine storm,^70^ increases the risk for activation of latent pulmonary histoplasmosis as reported in the case study.^71^ Of interest, pulmonary histoplasmosis and other fungal infections could potentially cause rehospitalization in recovered patients from Covid‐19 pneumonia, mainly in elderly subjects due to post‐Covid‐19 syndrome.^72^ Immune dysregulation in post‐Covid‐19 syndrome increases susceptibility to fungal infections. Besides, pulmonary histoplasmosis may develop after Covid‐19 pneumonia.^11^ A recent retrospective study involving 409 Covid‐19 patients showed that invasive fungal infections were associated with Covid‐19 severity and long hospitalization period in about 26.7%.^73^ Khanna et al.^74^ suggested that post‐Covid‐19 patients presented with persistent fever and abnormal lung radiological features could be localized pulmonary histoplasmosis. A narrative review indicated that invasive fungal infections including pulmonary histoplasmosis may complicate the clinical course of SARS‐CoV‐2 infection and are linked with high mortality mainly in critically ill patients admitted to the ICU.^75^

**Table 1:** The association between Covid‐19 and pulmonary histoplasmosis.

Study type	Findings	Reference
A cohort study	Anti‐H*. capsulatum* antibodies are present in 20.51% of Covid‐19 patients	Toscanini et al.^68^
A case study	Co‐infection with pulmonary histoplasmosis was detected in Covid‐19 patients.	Del Nogal et al.^70^
Review	Pulmonary histoplasmosis is linked with rehospitalization in recovered patients from Covid‐19.	Chavda et al.^72^
A retrospective study	Invasive fungal infections were associated with Covid‐19 severity.	Cattneo et al.^73^
A case study	Post‐Covid‐19 patients are associated with localized pulmonary histoplasmosis.	Khanna et al.^74^
A narrative review	Pulmonary histoplasmosis may complicate the clinical course of SARS‐CoV‐2 infection	Casalini et al.^75^
A case report study	Covid‐19 enhances the propagation of acute pulmonary histoplasmosis	deMacedo et al.^11^
A case report study and review	Pulmonary histoplasmosis is associated with SARS‐CoV‐2 infection	Messina et al.^76^

Pulmonary histoplasmosis may be worsened in Covid‐19 as pulmonary fungal infections were exaggerated during the SARS epidemic.^11^ In addition, patients with Covid‐19 and co‐infection with pulmonary fungal infections seem at higher risk for developing critical complications with high mortality.^77^ Different studies reported that Covid‐19 may increase the risk of developing pulmonary histoplasmosis due to immune dysregulation.^76^, ^78^, ^79^ In this state, de Macedo reported two cases of pulmonary histoplasmosis within 2 weeks of the development of Covid‐19 in Brazil without previous history of histoplasmosis.^11^ Both patients were positive for *H. capsulatum* DNA in sputum culture and were treated rapidly by itraconazole therapy.^11^ The authors suggest that Covid‐19 may enhance the propagation of symptomatic acute pulmonary histoplasmosis.^11^

The underlying causes for the development of acute pulmonary histoplasmosis in Covid‐19 could be related to lung injury and inflammation induced by Covid‐19.^76^ The use of corticosteroid treatment in Covid‐19 in severely affected Covid‐19 patients may also reactivate latent pulmonary histoplasmosis.^80^ Diagnosis of pulmonary histoplasmosis is difficult by culture and sensitivity, though molecular‐based and antigenic detection could be effective diagnostic methods.^81^ SARS‐CoV‐2 infection‐induced immune dysregulation could be the potential cause of the development of invasive fungal infections.^75^ Eibscutz et al.^82^ illustrated that positron emission tomography (PET) and computed tomography (CT) were of value in the detection of pulmonary and extra‐pulmonary complications as well as invasive pulmonary fungal infections in SARS‐CoV‐2 infection.^83^ In addition, PET is of great value in diagnosing lung pathologies in Covid‐19 patients.^83^

Furthermore, Basso^78^ and colleagues revealed the association between SARS‐CoV‐2 infection and pulmonary histoplasmosis in AIDS patients in a case‐report study. Pulmonary and/or disseminated histoplasmosis could be a possible fungal co‐infection in Covid‐19 patients with AIDS.^78^ Likewise, pulmonary histoplasmosis in AIDS patients with SARS‐CoV‐2 infection has been reported in Argentina.^76^ The real burden of the disease is still unknown due to the lack of epidemiological studies, notification and awareness of this fungal disease by many healthcare professionals as well as the availability of antigen tests or molecular detection assays for these fungi. For instance, there were no studies about nested PCR for Hc100 in respiratory specimens of critically ill patients with Covid‐19. It has been shown that disseminated histoplasmosis was found in 90% of the patient's kidney transplantations. The same proportion of patients had impaired transplant efficacy. In 60% of instances, the diagnosis was made a year after the transplant. Histopathology/culture‐based diagnosis had a 50% sensitivity rate, however nested PCR had a higher sensitivity and faster diagnostic speed.^84^ Only one study showed that surgical specimens with clinical suspicion of post‐Covid‐19 were positive for the Hc100 gene.^85^

These findings proposed that infection with SARS‐CoV‐2 could boost the danger of developing pulmonary histoplasmosis due to lung injury, inflammation, and the use of corticosteroid therapy in severely affected patients.

## 5 THE POTENTIAL LINK BETWEEN COVID‐19 AND PULMONARY HISTOPLASMOSIS

Lymphopenia is a hallmark of SARS‐CoV‐2 infection associated with Covid‐19 severity.^62^ In addition, T cell dysfunction promotes the pathophysiology of SARS‐CoV‐2 infection through prolonged antigen presentation to the auto‐reactive T cells with subsequent development of post‐Covid‐19 syndrome.^86^ Besides, disseminated pulmonary histoplasmosis is commonly developed in patients with idiopathic CD4 lymphocytopenia.^38^ Notably, defective cell‐mediated immunity is linked with the propagation of disseminated pulmonary histoplasmosis in AIDS patients, immunosuppressive agents, and chronic granulomatous diseases.^87^ Therefore, Covid‐19‐induced lymphopenia could potentially cause developing pulmonary histoplasmosis, chiefly in critically ill patients on corticosteroid therapy.

Furthermore, the T1h immune response is triggered in severe SARS‐CoV‐2 infection with subsequent macrophage activation and release of proinflammatory cytokines like IL‐1β, IL‐6, and TNF‐α.^88^ These immuno‐inflammatory changes increase the risk for the development of cytokine storm and associated MOF.^88^ In pulmonary histoplasmosis, dendritic and macrophages have significant roles in activating cellular pathways and releasing proinflammatory cytokines.^34^ Activated macrophages trigger the activation of the TH1 immune response and release of proinflammatory cytokines and IFN‐γ.^34^ During activation of the innate immune response, dendritic cells play a potential role in presenting antigens of *H. capsulatum* to the CD8 T cells, which have a central role in regulating cellular immunity during pulmonary histoplasmosis.^36^ Therefore, Covid‐19 patients with superimposed infection with *H. capsulatum* may augment the risk of poor clinical outcomes and mortality due to the exaggerated release of proinflammatory cytokines.

Moreover, impairment of IFN‐γ release and function in *H. capsulatum* infection amplifies the risk for the progress of severe complications.^42^ Patients with mutations in IFN‐γ receptors had an increased risk for the advancement of severe *H. capsulatum* infection.^43^ In Covid‐19, there is a noteworthy functional deficiency of IFN‐γ response.^89^ SARS‐CoV‐2 can evade the antiviral effects of IFN‐γ, which is regarded as a first‐line defense against different viral infections, including SARS‐CoV‐2.^89^ As well, SARS‐CoV‐2 proteins can directly inhibit IFN‐γ response. Thus, inhibition of IFN‐γ response in both SARS‐CoV‐2 and *H. capsulatum* infections impair innate immune response and immunological interaction with adaptive immune response causing hyperinflammation and exaggerated immune response. Thus, inhaled INF‐1β could be effective in the treatment of severe Covid‐19.^90^, ^91^

Moreover, HSP60, which regulates intracellular protein folding, is regarded as an important surface molecule that mediates phagocytosis of *H. capsulatum* by the activated macrophages.^21^ HSP60 acts as a ligand for macrophage receptors during fungal infection, though this interaction induces a mild host immune response.^22^ In this state, *H. capsulatum* replicates and survives within the host cells. HSP60 of *H. capsulatum* protects against antibody response and improves biofilm formation.^21^ Heine, HSP60, which is essential for macrophage activation and release of proinflammatory cytokines, is activated during *H. capsulatum* infection causes intense inflammatory disorders. Remarkably, *H. capsulatum* infection can induce the expression and release of proinflammatory cytokines, chiefly IL‐6 and IL‐8, in lung epithelial cells through the induction expression of integrin and its membrane interaction.^92^ Besides, in SARS‐CoV‐2 infection, HSP60 is triggered, leading to hyperinflammation and development of cytokine storm via activation of TLR4 and nuclear factor kappa B.^93^ Thus, *H. capsulatum* infection may increase the severity of Covid‐19 through the HSP60‐dependent pathway.

Of note, reducing Treg and over‐activation of Th17 may modulate the pathogenicity of fungal infections, including *H. capsulatum*.^40^ The reduction of circulating Treg is also associated with the acceleration of *H. capsulatum* infection.^40^ In Covid‐19, Tregs are reduced significantly leading to an exaggerated immune response and the development of immuno‐inflammatory complications.^94^ Th17 activation is linked with immune activation and the release of proinflammatory cytokines.^95^ Notably, Treg exerts inhibitory effects on CD8 and CD4 with subsequent attenuation release of proinflammatory cytokines and development of hypercytokinemia.^94^ Therefore, Th17 activation and reduction of Treg are associated with increasing severity of both *H. capsulatum* and SARS‐CoV‐2 infections.

On the other hand, different inflammatory signaling pathways are activated in both *H. capsulatum* and SARS‐CoV‐2 infections. For example, the NLRP3 inflammasome, which augments the inflammatory and immune response to SARS‐CoV‐2 infections, is highly activated leading to the propagation of cytokine storm.^96^ Pan et al.^97^ illustrated that the SARS‐CoV‐2 N protein activates NLRP3 inflammasome with subsequent hyperinflammation in severely affected Covid‐19 patients and experimental mice. Similarly, *H. capsulatum* infection activates the expression of IL1β and caspase‐1 via stimulation of NLRP3 inflammasome.^98^ Thus, Covid‐19 with *H. capsulatum* co‐infection may exacerbate the immuno‐inflammatory disorders causing life‐threatening complications and poor clinical outcomes.

Moreover, NADPH‐oxidase and p38MAPK are activated in both *H. capsulatum* infection and Covid‐19, leading to oxidative stress and hyperinflammation, respectively.^42^, ^99^, ^100^ Therefore, Covid‐19 with *H. capsulatum* co‐infection may lead to oxidative stress and hyperinflammation propagation. Furthermore, Heine, antioxidant and anti‐inflammatory agents may reduce the severity of Covid‐19 and/or pulmonary histoplasmosis. Finally, the mechanistic target of rapamycin (mTOR) is highly activated in pulmonary histoplasmosis and Covid‐19 leading to induction apoptosis, autophagosomes, and propagation of hyperinflammation.^101^, ^102^, ^103^

**Figure 3:**
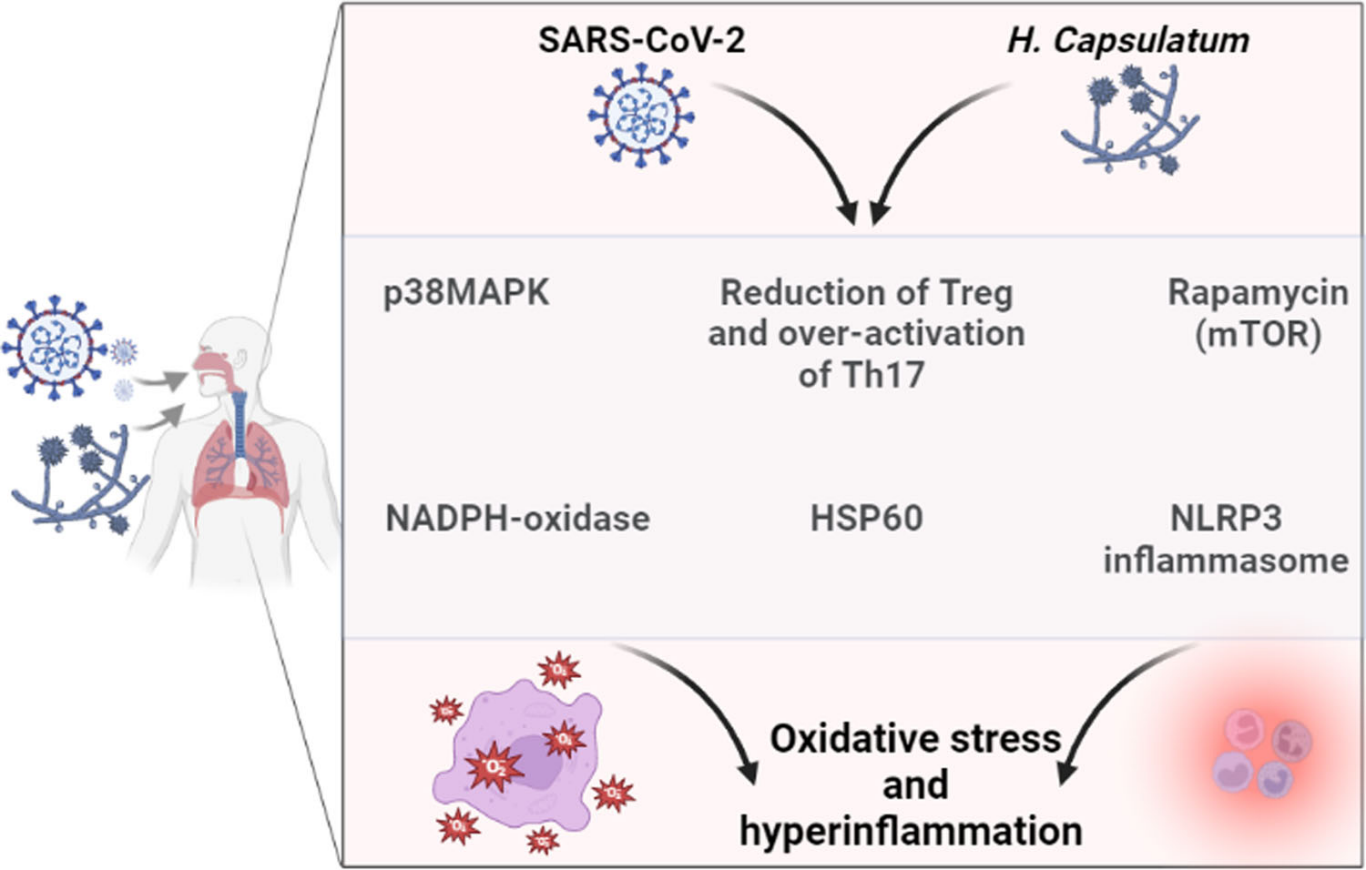
The inflammatory signaling in Covid‐19 and pulmonary histoplasmosis.

Taken together, activation of inflammatory signaling pathways during *H. capsulatum* infection may increase the severity of Covid‐19 and vice versa (Figure 3). The present review had several limitations, including the scarcity of prospective clinical studies concerning the development of pulmonary histoplasmosis in Covid‐19 patients. Also, the molecular and mechanistic role of *H. capsulatum* infection was not well illustrated in SARS‐CoV‐2 infection. However, the present review revealed the possible link between Covid‐19 and pulmonary histoplasmosis for the first time.

## 6 CONCLUSIONS

Covid‐19 may increase the risk of developing pulmonary histoplasmosis due to lung injury, inflammation, and the use of corticosteroid therapy in severely affected patients. Pulmonary histoplasmosis is common in immune‐comprised patients; therefore, immune dysregulation, chiefly lymphopenia in Covid‐19, may increase the risk for the progress of pulmonary histoplasmosis. Furthermore, different inflammatory signaling pathways are also activated in both *H. capsulatum* and SARS‐CoV‐2 infections. Thus, activation of inflammatory signaling pathways during *H. capsulatum* infection may increase the severity of Covid‐19 and vice versa. In this state, preclinical and clinical studies are warranted in this regard.

## AUTHOR CONTRIBUTIONS

**Hebatallah M. Saad and Ali I. Al‐Gareeb**: conceptualization, data collection, writing of the manuscript and responding to reviewers’ comments. **Yahya A. Almutawif, Athanasios Alexiou, Marios Papadakis, Hamza M. A. Eid, and Gaber El‐Saber Batiha**: writing, supervision, editing of the manuscript and responding of reviewer's comments. **Hebatallah M. Saad**: preparation of figures and editing of the manuscript. All authors read and approved the final version of the manuscript.

## CONFLICT OF INTEREST STATEMENT

The authors declare no conflicts of interest.

## Data Availability

Not applicable.
